# Combination of Five Body Positions Can Effectively Improve the Rate of Gastric Mucosa's Complete Visualization by Applying Magnetic-Guided Capsule Endoscopy

**DOI:** 10.1155/2016/6471945

**Published:** 2016-11-29

**Authors:** Yuting Qian, Sheng Wu, Qi Wang, Lumin Wei, Wei Wu, Lifu Wang, Ye Chu

**Affiliations:** Department of Gastroenterology, Shanghai Jiaotong University Medical School Affiliated Ruijin Hospital, 197 Second Ruijin Road, Shanghai 200025, China

## Abstract

*Objectives*. Achieving a comprehensive view of gastric mucosa has been a challenge for magnetic-guided capsule endoscopy (MGCE) for years. This study works on optimizing the performance of MGCE by changing the conventional positions to the five body positions.* Methods*. Sixty patients were enrolled in the study and underwent MGCE. All patients were asked to adopt five body positions (left lateral, supine, right lateral, knee-chest, and sitting). In each position, the ability to visualize the six gastric landmarks (cardia, fundus, body, angulus, antrum, and pylorus) was assessed. Rates of complete visualization were calculated for different position combinations.* Results*. Supine position was the best for cardia and body visualization (91.7% and 86.7%, resp., *p* < 0.001). Left lateral position was the best for fundus visualization (91.7%, *p* < 0.001). Knee-chest position was the best for angulus observation (80.0%, *p* < 0.001). Right lateral and sitting positions were the best for antrum observation (88.3% and 90.0%, resp., *p* < 0.001). Right lateral position was the best for pylorus observation (81.7%, *p* < 0.001). The supine + right lateral + knee-chest combination achieved better angulus visualization than conventional 3-position combination (93.3% versus 63.3%, *p* < 0.001). Five-position combination significantly improved the comprehensive gastric landmark visualization (93.3%, *p* < 0.001).* Conclusion*. Compared with 3-position combination, 5-position combination should be adopted for gastric mucosal visualization by MGCE.

## 1. Introduction

During the last 30 years, esophagogastroduodenoscopy (EGD) has been recognized as the “gold standard” for the diagnosis of gastric pathology, in which biopsy and treatment can be performed at the same time. However, the application of EGD in screening, investigation, and monitoring of gastric disease is limited due to the unpleasant intubation experience. Additionally, sedation used during the procedure comes with high cost and sedation-related risks [1–3]. So, noninvasive methods for detecting gastric lesions are needed. Since 2001, when the American Food and Drug Administration approved wireless capsule endoscopy, the new method has become a diagnostic tool for small-bowel examination, being known for its feasibility, safety, and high detection rate [4, 5]. Moreover, capsule endoscopies designed for the esophagus and colon have shown promising outcomes [6, 7]. Recently, capsule endoscopy designed for stomach was developed and several kinds of magnetic-guided capsule endoscopy (MGCE) for clinical use showed benefits [8–10].

Nowadays, the left lateral + supine + right lateral position combination which can facilitate the capsule's navigation in the stomach is used as “routine” during MGCE examination. But achieving the comprehensive view of gastric mucosa remains a challenge due to the special anatomical structure of the stomach. In clinical practice, we found that reposition could optimize the performance of MGCE. Until now, there is no report focusing on the use of position combination, neither the standard of practice for position combinations during MGCE examination.

This study introduced five positions (left lateral, supine, right lateral, knee-chest, and sitting) during the examination which could optimize the comprehensive gastric visualization of MGCE in human stomach. Each position was evaluated by the rate of comprehensive visualization of six gastric landmarks: gastric cardia, fundus, body, angulus, antrum, and pylorus.

## 2. Materials and Methods

### 2.1. Study Cohorts

**Figure 1:**
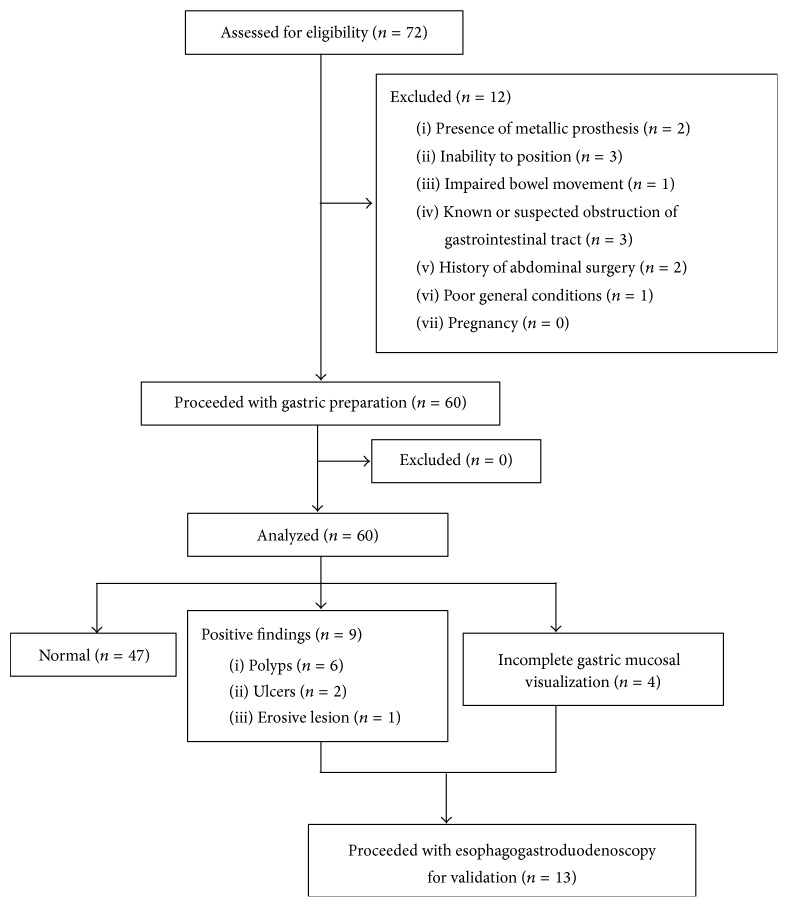
Flow chart of the study.

Approval for this study was granted by the ethics committee of Shanghai Jiaotong University Medical School Affiliated Ruijin Hospital. Seventy-two patients enrolled for MGCE examination at our outpatient clinic between May 2015 and February 2016 were registered. Indications included abdominal pain, diarrhea, anorexia, and anemia with suspected gastrointestinal bleeding. Some of the patients were actually healthy volunteers. The exclusion criteria were the presence of metallic prosthesis, inability to position specially for old or debilitated patients, impaired bowel movement, known or suspected obstruction of gastrointestinal tract, history of abdominal surgery, poor general conditions, and pregnancy. Cases with qualified gastric preparation which was defined as good cleanliness (fluid was transparent and less than 5% mucosa was covered by stomach contents) and good visualization (more than 75% mucosa was observed) were included for this study. This study finally selected sixty patients to analyze. All subjects were aware of the procedure and MGCE-related risks. All subjects signed consent forms. Patients with positive findings or incomplete gastric visualization were suggested to undergo EGD for validation (Figure 1). All subjects were followed up for two weeks to estimate MGCE complications.

### 2.2. Magnetic-Guided Capsule Endoscopy Technique

The MGCE system used in this study is Navi-Cam (Ankon Technologies Co. Ltd., Wuhan, China). ANKON Navi was approved by the Chinese Food and Drug Administration in 2013 and was proven to be safe and feasible for the examination of the human stomach [9, 11].

The MGCE system consists of capsule endoscopy, a guidance magnetic robot, a data recorder, and a computer workstation equipped with software for real-time observation and manipulation. The endoscopic capsule is 28 × 12 mm in size with a permanent magnet in one dome and a complementary metal-oxide-semiconductor (CMOS) image sensor in the other dome. The CMOS image sensor captures images at 2 frames per second with a field of 140° view. All images were transmitted into the workstation simultaneously and were captured in real time. The capsule is operated by a C-arm type guidance magnetic robot which has five motions (two rotational and three translational) and two manipulation modes (joystick mode and automatic mode). The magnetic field can be adjusted to reach a maximum of 200 mT. For general use, 5 mT to 30 mT is enough. With the guidance magnetic robot and magnetic field, capsules could perform linear or rotational movements for better visualization of gastric mucosa.

### 2.3. Procedure

**Figure 2:**
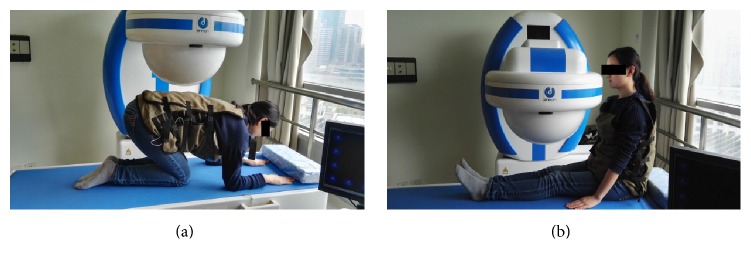
Demonstration of KC and Si positions. (a) KC position. (b) Si position. KC: knee-chest; Si: sitting.

Subjects had overnight fasting (>8 h) and were given 500 ml clear water 2-3 hrs before the examination. Metal items were removed before the procedure. 10 ml Simethicone (Berlin-Chemie AG), 20000 units of Pronase Granules (Beijing Tide Pharmaceutical Co. Ltd.), and 5 g of Sodium Bicarbonate (Beijing Tide Pharmaceutical Co. Ltd.) were diluted in 500 ml clear room temperature water and were given 60 min before the examination for better capsule navigation. Image-receiving device was attached to patients lying inside the magnetic field. After 500–600 ml water was ingested for stomach distention, a subject swallowed capsule endoscopy together with 5 ml clear water in an upright position. In addition to the conventional three exanimation positions (left lateral (L), supine (Su), and right lateral (R)), subjects were asked to change to knee-chest (KC, Figure 2(a)) and finally sitting (Si, Figure 2(b)) positions.

### 2.4. Main Outcome Measures

Visualization of primary gastric anatomical landmarks (cardia, fundus, body, angulus, antrum, and pylorus) was recorded in real time. Comprehensive visualization was defined as 100% mucosal visualization of all 6 landmarks. Partial viewed image or unclear image was labeled as “not viewed.” Two endoscopists with experience of reading more than 200 capsule endoscopy cases were chosen for this study and were blinded to each other's results. If they reached different results, they had a discussion until agreement was achieved.

### 2.5. Statistical Analysis

Continuous variables are presented as mean ± SD and categorical variables are presented as frequency (%). Statistics with their 95% confidence intervals (CI) are given. Pearson chi-square test was used to analyze the complete mucosal visualization rate for different positions. *p* value of less than 0.05 was considered statistically significant. All analyses were carried out using SPSS version 20 and Microsoft Excel 2010 (IBM Corp., Armonk, New York, USA).

## 3. Results

### 3.1. Study Population

**Figure 3:**
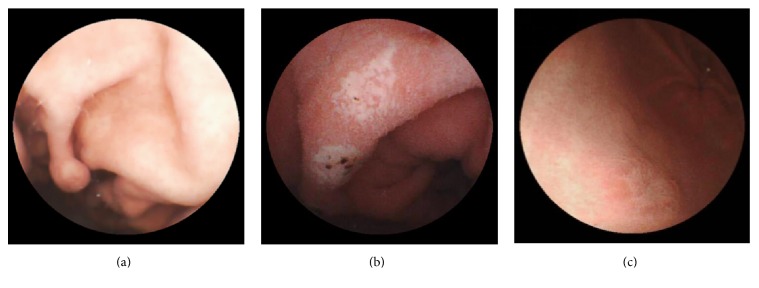
Positive findings in the stomach from different body positions. (a) A polyp located in the gastric body from Su position. (b) Multiple ulcers located in the antrum from R position. (c) An ulcer located in the antrum from Si position. L: left lateral; Su: supine; R: right lateral; KC: knee-chest; Si: sitting.

Sixty subjects were finally included and analyzed in the study; 28 (46.7%) were males. The average age was 43.1 ± 15.4 years. Indications for MGCE were abdominal pain (41.7%), diarrhea (1.7%), anorexia (3.3%), and anemia with suspected gastrointestinal bleeding (3.3%), along with healthy volunteers (50%). All capsules were excreted in two weeks, and complications such as capsule retention, obstruction, or inhalation were not observed. The average exam time was 42.0 ± 20.3 min. Among positive findings, polyps located in the gastric body were found in 4 cases and polyps located in the fundus were found in 2 cases from Su (Figure 3(a)), L, and KC positions. A case with multiple ulcers located in the antrum was found from R and Si position (Figure 3(b)). A case with an ulcer located in the antrum was found from Si position (Figure 3(c)). Single erosive lesion located in the antrum was found from L position in another case. Patients with positive findings and incomplete gastric mucosal visualization were suggested to undergo EGD. Positive findings mentioned before were all observed by EGD and no further lesions were visualized by EGD. Then, patients with positive findings were all followed up with systemic therapies. Seven cases reached different results, and, after rereading and discussions, agreements were achieved by two endoscopists.

### 3.2. Gastric Mucosal Visualization from Single Positions

**Figure 4:**
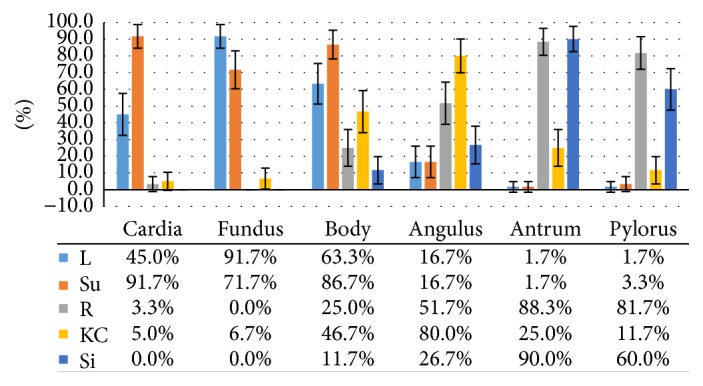
Comparison of gastric mucosal visualization rates from single positions. L: left lateral; Su: supine; R: right lateral; KC: knee-chest; Si: sitting.

In this study, complete visualization of all 6 landmarks was not achieved from any single position.  Figure 4 showed the gastric mucosal visualization rates from different positions. Su position was the best for cardia visualization (91.7%, 95% CI: 84.7%–98.7%, *p* < 0.001). L position was the best for fundus visualization (91.7%, 95% CI: 84.7%–98.7%, *p* < 0.001). Su position was the best for body visualization (86.7%, 95% CI: 78.1%–95.3%, *p* < 0.001). KC position was the best for angulus observation (80.0%, 95% CI: 69.9%–90.1%, *p* < 0.001). R position and Si position were the best for antrum visualization [88.3% (95% CI: 80.2%–96.5%) and 90.0% (95% CI: 82.4%–97.6%), resp., *p* < 0.001]. And R position was the best for pylorus observation (81.7%, 95% CI: 71.9%–91.5%, *p* < 0.001).

### 3.3. Complete Mucosal Visualization Rate from All Position Combinations

**Figure 5:**
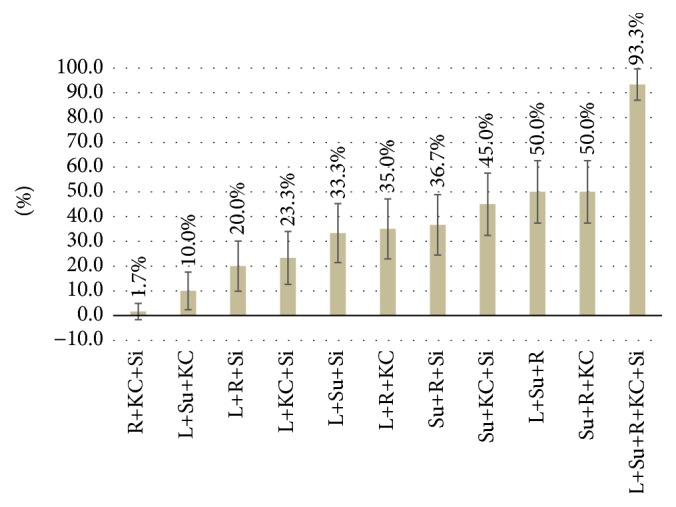
Comparison of complete visualization rates of different position combinations. L: left lateral; Su: supine; R: right lateral; KC: knee-chest; Si: sitting.

Figure 5 showed the rates of comprehensive mucosal visualization in all position combinations (3- and 5-position combination included). Maximal rate of comprehensive mucosal visualization was achieved by the combination of all five positions as compared with other combinations (93.3%, 95% CI: 87.0%–99.6%, *p* < 0.001). Four cases with incomplete mucosal visualization had deficiency in pylorus observation. Slight chronic superficial gastritis was verified in all these four participants after EGD examination.

### 3.4. Complete Mucosal Visualization from 3-Position Combinations

**Figure 6:**
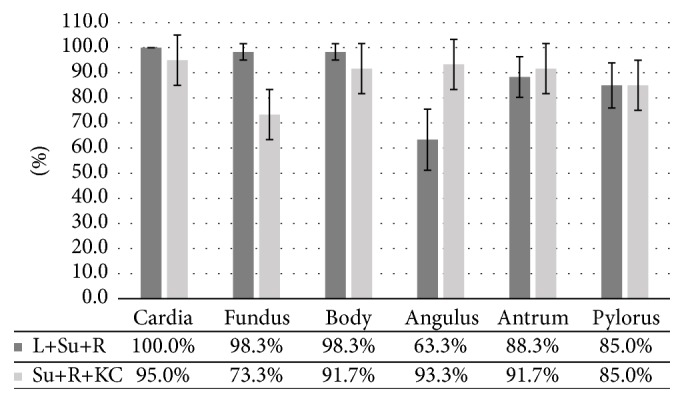
Comparison of complete visualization of anatomical landmarks from Su+R+KC and L+Su+R combinations. L: left lateral; Su: supine; R: right lateral; KC: knee-chest; Si: sitting.

All kinds of 3-position combinations were analyzed in this study (Figure 5). The complete visualization rates from L+Su+Si, L+R+KC, Su+R+Si, Su+KC+Si, L+Su+R, and Su+R+KC combinations had no significant differences (*p* = 0.210) which were significantly better than the other combinations (*p* < 0.001).  Figure 6 showed the complete visualization of gastric landmarks from L+Su+R and Su+R+KC combinations which had better complete visualization rates (50.0% and 50.0%, resp.). For the fundus observation, L+Su+R combination was significantly better than Su+R+Si combination [98.3% (95% CI: 95.1%–101.6%) versus 73.3% (95% CI: 62.1%–84.5%), *p* < 0.001]. Regarding the angulus visualization, Su+R+Si combination was significantly better than L+Su+R combination [93.3% (95% CI: 87.0%–99.6%) versus 63.3% (95% CI: 51.1%–75.5%), *p* < 0.001]. There was no significant difference between the 2 combinations in the cardia, body, antrum, and pylorus visualization.

## 4. Discussion

Since the idea of driving capsule endoscopy controlled by magnetic field appeared in 2006 [12], the magnetic-guided capsule endoscopy has become a hot topic. The feasibility of several kinds of MGCEs manipulated* in vivo* and in volunteers has been reported in early years [10, 13–15]. As the MGCE technique improves, it showed similar diagnostic value of gastric lesions compared to traditional EGD [11, 16–18]. However, optimal gastric mucosal visualization has still been a challenge for MGCE. Incomplete visualization is usually caused by poor stomach cleanliness, mucosal resistance, excessive stomach motility, poor gastric distention, and early gastric passage [19]. The experience of operators and patients' position changes are also important factors.

To optimize the performance of MGCE, Rahman et al. [20] used multiplanner CT modeling to determine the better positioning of MGCE in stomach. The placement of MGCE was held in 5 gastric stations by a manual-controlled magnet, and the locations of capsule were detected by CT modeling. In their study, station combinations are needed for better visualization. This suggested that the change of capsule positioning at certain gastric landmarks could increase the complete visualization rate. Due to the lack of manual-controlled magnet and CT-guided localization, the magnetic field for MGCE was unable to drive the capsule precisely to the strategic stations as mentioned in their study.

In light of this observation, we were in search of other ways to facilitate capsule navigation and simplify capsule movement at the same time. After repeated trials, we found that change of subject positions facilitated capsule navigation upon adequate distention of the stomach with water, which resulted in better gastric mucosal visualization, especially for some gastric landmarks that are difficult to observe such as angulus and pylorus. In this way, both close and remote mucosal visualization were easily achieved. So far, there is no report on the position change during MGCE examination.

Since Rey et al. [10] introduced the L+Su+R combination in MGCE practice with promising gastric visualization quality, this position combination has been adopted as “routine” worldwide. In their study, the complete visualization rates of 4 gastric landmarks (the cardia, fundus, body, and antrum) were 75%, 73%, 96%, and 98%, respectively. Two years later, the same group carried out another blinded comparison trial [16] with the same position combination, and the observation rates of 5 gastric landmarks (the cardia, fundus, body, antrum, and pylorus in addition) were reported as 88.5%, 85.2%, 93.4%, 86.9%, and 88.5%, respectively. The difference in visualization rates between the two reports might result from the improvement of image-reading technique, larger sample size (53 in the former study and 71 in the latter), change of subject characteristics (healthy volunteers were included in the former study but the latter one only recruited patients with gastric pain), shortened examination time (30.0 min versus 17.4 min), and different success rates of the procedure (98% versus 85.9%). Compared with the results of Rey et al. [10, 16], the higher cardia and fundus visualization rates in our study might be due to better gastric cleanliness and distention which were achieved by using Simethicone, Pronase Granules, and Sodium Bicarbonate for gastric preparation.

**Figure 7:**
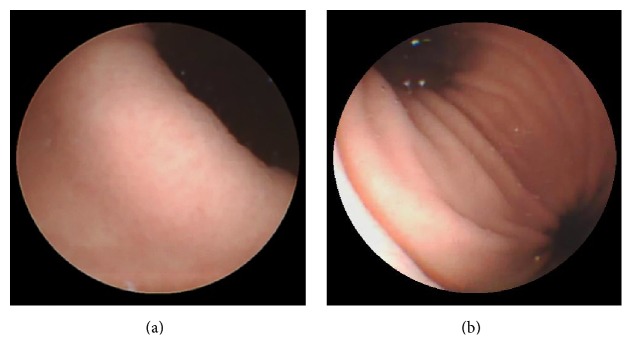
Angulus visualization from different positions. (a) Angulus visualization from Su position. (b) Angulus visualization from KC position. Su: supine; KC: knee-chest.

In clinical practice, complete visualization of angulus was usually partially achieved (Figure 7(a)) due to its obscure anatomical location. Observation of its movement and the vertical part of the angulus is especially inadequate. However, the angulus is an important part involving malignant lesions that can be easily missed endoscopically. To address this problem, we tried the KC position from which the capsule moves slightly towards the angulus by gravity. In this study, the view on MGCE was similar to the view on the inverted EGD. As a result, KC position showed significantly higher complete angulus observation rate than any other positions. Additionally, from this position, the movement of the angulus was captured and close mucosal observation was achieved (Figure 7(b)). This might explain the significantly higher angulus visualization rate of the Su+KC+Si combination compared with the conventional L+Su+R combination. The Si position was also introduced in our study. From this position, MGCE reached the bottom of antrum by gravity, which facilitated the observation of antrum and pylorus. Therefore, Si position is another option for cases with inadequate antrum and pylorus observation from R position. Overall, the 5-position combination significantly improved the implement of complete gastric landmark visualization. Four cases with incomplete gastric view were caused by a strong pyloric contraction during the procedure.

In this study, several limitations existed. First, participants with negative MGCE findings have not undergone conventional gastroscopy to verify findings due to the fear of EGD intubation which might lead to certain missed diagnosis. Second, larger sample size was required to identify the characteristics of each combination. Third, the fact that a large number of healthy volunteers were enrolled might decrease the positive findings and simplify the operating procedure. Fourth, old and debilitated patients were excluded from this study due to the fact that knee-chest position was tough or even dangerous, so better ways need to be further explored.

In conclusion, all 5 positions adopted for the MGCE examination in this study showed their own advantages in gastric mucosal visualization. The supine + right lateral + knee-chest combination and the conventional left lateral + supine + right combination had similar complete visualization rates, but the former one showed better angulus visualization. The 5-position combination achieved the highest rate of complete gastric landmark visualization. Compared with conventional 3-position combination, the 5-position combination should be adopted for gastric mucosal visualization by MGCE.
